# Intraperitoneal ampicillin treatment for peritoneal dialysis- related peritonitis with *Listeria monocytogenes* – a case report

**DOI:** 10.1186/s12882-020-02068-1

**Published:** 2020-09-18

**Authors:** Kristina Boss, Ina Wiegard-Szramek, Jan Dziobaka, Andreas Kribben, Sebastian Dolff

**Affiliations:** 1 Department of Nephrology, University Hospital Essen, University-Duisburg Essen, 45147 Essen, Germany; 2 Institute of Medical Microbiology, University Hospital Essen, University-Duisburg Essen, Essen, Germany; 3 Department of Infectious Diseases, University Hospital Essen, University-Duisburg Essen, Essen, Germany

**Keywords:** Listeria, Peritoneal dialysis, Peritonitis, Ampicillin, Case report

## Abstract

**Background:**

Peritoneal dialysis (PD)-related peritonitis is a rare but serious complication and is associated with increased morbidity and mortality rates. It is most commonly caused by *Staphylococcus aureus* or *Staphylococcus epidermidis*, but infection with *Listeria monocytogenes* may also occur. Recommendations for antibiotic treatment of a Listeria infection are currently based on a small number of case reports and suggest the administration of ampicillin. But unlike vancomycin or gentamicin, for ampicillin the route of application, the dosage, and the duration of treatment have not yet been established. We report a case in which PD-associated peritonitis due to Listeria infection was treated with ampicillin administered intravenously and intraperitoneally, separately and in combination.

**Case presentation:**

A 72-year-old man with chronic kidney disease stage 5 dialysis (CKDG5D) secondary to hypertension and diabetes was hospitalised in April 2020 because of PD-related peritonitis caused by a Listeria infection. In accordance with the results of resistance tests, the patient was treated with intravenous ampicillin at a dosage of 6 g twice daily. After initial treatment the leukocyte count in the PD effluent had decreased substantially, but it was permanently reduced only with the addition of intraperitoneal ampicillin (4 g daily). Efficient serum concentrations of ampicillin were determined for both routes of administration, intravenous and intraperitoneal.

**Conclusion:**

This is the first case report demonstrating that PD-related peritonitis due to *Listeria monocytogenes* infection can be treated with intraperitoneal ampicillin and monitored by the determination of peripheral serum concentrations of ampicillin.

## Background

Peritoneal dialysis (PD) is an established method of renal replacement therapy. PD-related peritonitis is a rare but serious complication and is associated with increased morbidity and mortality rates. In addition, each instance of peritonitis reduces the transport capacity of the peritoneum and eventually limits the effectiveness of dialysis [1].

PD-related peritonitis is most commonly caused by gram-positive bacteria, such as *Staphylococcus epidermidis* or *Staphylococcus aureus* [2], but infection with *Listeria monocytogenes* may also occur [3]. Antibiotics used to treat peritonitis may be administered intravenously, orally, or intraperitoneally, but intraperitoneal administration is preferred to ensure maximal antibiotic concentrations at the site of infection [2]. Recommendations of antibiotic therapy for Listeria infection are currently based on a small number of case reports and suggest the administration of ampicillin. But, unlike vancomycin or gentamicin, for ampicillin the route of application, dosage, and duration of treatment have not yet been established [2]. We report a case of PD-associated peritonitis due to *L. monocytogenes* infection that was managed with ampicillin administered intravenously and intraperitoneally, separately and in combination.

## Case presentation

A 72-year-old man with chronic kidney disease stage 5 dialysis (CKDG5D) secondary to hypertension and diabetes was hospitalised in April 2020, reporting abdominal pain and fever after noticing cloudy PD effluent 3 days before hospitalisation.

The initial physical examination showed that the patient’s general condition was reduced and that he was obese (body mass index [BMI], 34.5). He had a fever of 38.5 °C. His blood pressure was 116/74 mmHg, his heart rate was 80 beats per minute, his respiratory rate was 14 breaths per minute, and his oxygen saturation was 96% without supplementation. He reported no vomiting, nausea, or diarrhoea. There were no signs of meningeal irritation. Examination showed strong, diffuse abdominal tenderness with maximal pain in the right lower abdomen. The PD catheter exit site was clean and dry, and there was no tunnel tenderness.

The patient’s medical history included type 2 diabetes (first diagnosed in 2012, insulin-dependent since 2018), hypertension, severe coronary heart disease requiring multiple revascularizing procedures (including bypass surgery), and persistent atrial fibrillation necessitating implantation of a cardiac pacemaker and a left atrial appendage (LAA) occluder. The patient had required continuous ambulatory peritoneal dialysis (CAPD) since June 2018 and had not experienced any previous dialysis-related infections or other complications.

During the course of this infection, an outpatient analysis of the PD effluent found a leukocyte count of 11,000/nL. At the time of hospital admission, the first PD effluent was cloudy, with a leukocyte count of 7060/nL. Initial blood tests found elevated levels of indicators of inflammation: C-reactive protein (CRP), 17.5 mg/dL (normal, < 0.5 mg/dL); procalcitonin (PCT), 3.22 ng/mL (normal, < 0.5 ng/mL). Blood gas analysis found a serum lactate level of 2.4 mmol/L (normal, < 1.2 mmol/L) but no acidosis or electrolyte disorders. The urine test strip results were negative for leukocytes and nitrites.

Initially, we intensified dialysate exchanges till PD effluent was clear and the patient’s abdominal pain decreased followed by an increased number of daily dialysate exchanges for the first 3 days of treatment. From the fourth day of treatment on, the patient was placed on a continuous cyclic peritoneal dialysis (CCPD) regimen.

After obtaining blood, urine, and PD effluent samples for microbiological analysis, we initiated antibiotic therapy because analysis of ambulatory PD effluent detected L. monocytogenes. In the first 24 h of hospital stay the patient showed fever up to 39 °C and hypotension associated with reduced vigilance. Therefore, a septic course of disease in terms of a meningitis could not be ruled out. On the basis of the results of resistance testing, we treated the patient with ampicillin administered intravenously at a dosage of 6 g twice daily according to German meningitis guideline [4].

We also treated the patient empirically with intraperitoneal cefazolin and ceftazidime because we could not rule out infection with a different or an additional bacterium at the time of admission.

Microbiological cultures of the PD effluent samples obtained on the first day of hospitalisation detected L. monocytogenes. The German Consultant Laboratory for Listeria confirmed the identification and classified the bacterium as a member of serotype IIa. Additional microbiological analysis confirmed the presence of L. monocytogenes in the PD effluent. Because no other pathogen was detected in the PD effluent, intraperitoneal antibiotic treatment with cefazolin/ceftazidime was discontinued after the administration of a single dose on the first day of treatment. No bacterial growth was detected in cultures of blood or urine samples. Cultures of consecutive samples of PD effluent obtained on the next day and on ten other days during the hospital stay did not grow bacteria.

During intravenous antibiotic treatment, the leucocyte count in the PD effluent decreased continually to 315/nL, but on the fifth day of treatment the leukocyte count increased to 3778/nL. The CCPD regimen had not changed, and the CRP and PCT levels had decreased continually. There were no further episodes of fever. To rule out underdosing of ampicillin, we determined the serum ampicillin level and its association with the minimal inhibitory concentration (MIC) of the Listeria isolate, which was determined to be 0.5 mg/L and was interpreted as “susceptible” according to the clinical breakpoints provided by the European Committee on Antimicrobial Susceptibility Testing (EUCAST) [5]. With expert consent, we determined to establish a therapeutic range of serum ampicillin levels of at least four times the MIC [5]. The first test of the serum ampicillin level (after 5 days of intravenous ampicillin administration with the last dose given 7 h before sample taking) found a concentration of 149 mg/L. We collected further samples at two and 4 h after the next dose of intravenous antibiotic, and tests showed sufficient serum ampicillin levels of 235 mg/L at 2 h and 95.5 mg/dL at 4 h. Therefore, we continued the previous intravenous antibiotic treatment.

At day seven the leukocyte count in the PD effluent had increased to 5182/nL; therefore, escalation of antibiotic therapy became crucial. The recent International Society for Peritoneal Dialysis (ISPD) Guideline does not provide any recommendations for monotherapy with intraperitoneal ampicillin by intermittent application (with one exchange daily) [2]. Also, the adequate duration of therapy has not yet been established. We decided that, in addition to continuing intravenous administration of ampicillin, we would add a dose of 4 g ampicillin to a 2-L bag of Extraneal dialysate (Baxter International, Deerfield, IL, USA). The bag was shaken to ensure adequate mixing of the drug in the dialysate. The antibiotic-containing dialysate was drawn into the peritoneal cavity and allowed to dwell for 12 h.

**Fig. 1:**
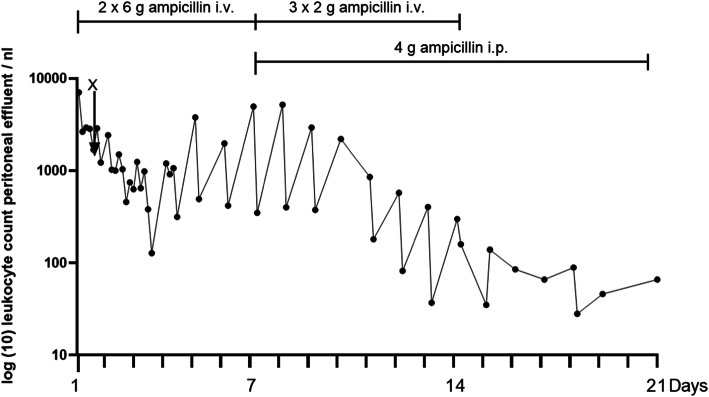
Leukocyte count in peritoneal dialysis effluent over the course of treatment for a patient with peritoneal dialysis–related peritonitis. Figure shows the course of the leukocyte count in the peritoneal dialysis effluent leukocytes over the 21-day treatment period. X marks single administration of 1 g ceftazidime/1 g cefazolin. All other information about antibiotic dosing refers to daily administration, only intravenous ampicillin application day 1–6, combined intravenous and intraperitoneal application day 7–14, only intraperitoneal application day 15–21. i.v., intravenous; i.p., intraperitoneal

With this combination of intravenous and intraperitoneal antibiotic therapy, the leukocyte count in the PD effluent finally declined permanently. We continued drug monitoring and detected sufficient serum ampicillin levels even when the drug was administered intraperitoneally only at a dosage of 4 g daily. The leukocyte count stayed low. Detailed information about antibiotic therapy and serum ampicillin levels can be found in the figure and the table (Table 1 and Fig. 1).

**Table 1:** Serum levels of ampicillin over the course of treatment for a patient with peritoneal dialysis–related peritonitis

Day of Treatment	**Method of application**	Time point	Serum ampicillin level [mg/l]
5	intravenous	0 h	149
		2 h	235
		4 h	96
14	Intravenous	0 h	165
		2 h	241
		4 h	181
15	intraperitoneal	0 h	53
		2 h	97
		4 h	110
18	intraperitoneal	0 h	25
		2 h	65
		4 h	85
		8 h	91

After the PD leukocyte count had been lower than 100/nL for 7 days, we discontinued antibiotic therapy on day 21 and discharged the patient from the hospital. A more detailed anamnesis led us to suspect that the Listeria infection was caused by consumption of a creamed cheese. This cheese was analysed by the local health authority, but no Listeria could be detected. Moreover, the bacterium was not detectable in stool samples obtained during inpatient treatment. Therefore, the origin of the infection remained unclear. Outpatient examination 3 days after hospital discharge found that the patient was fit and well. There were no signs of a relapse of infection. The patient is under regular outpatient supervision. Within 3 months there was no recurrence of infection.

## Discussion and conclusion

*L. monocytogenes* can cause PD-related peritonitis and is associated with a mortality rate as high as 30% in cases of systemic infection [6]. In recent years, a few case reports of PD-related peritonitis caused by Listeria have been published [7, 8], but the treatment strategies mentioned in these reports varied. Antibiotic treatment involved ampicillin, vancomycin, or aminoglycosides as monotherapy or in various combinations, and the drugs were administered intravenously, intraperitoneally, orally, or in some combination of these routes. However, these reports did not recommend a standardized therapy. For several of these cases treatment with vancomycin was not successful, and the infection could be effectively treated only with ampicillin. Overall, it therefore seemed reasonable to begin with ampicillin as a first-line therapy, with regard to a possible systemic infection such as meningitis. On the basis of these reports, we decided to treat our patient with intravenous ampicillin. During the first days of treatment the leukocyte count in the PD effluent decreased substantially, but it was permanently reduced only after the addition of intraperitoneal ampicillin.

Pharmacokinetic studies have investigated the toxicity of aminoglycosides, such as gentamicin or tobramycin, for patients undergoing PD, but no such studies have been performed for ampicillin [9]. Although Lorenzen et al. performed a pharmacokinetic analysis of ampicillin among patients undergoing hemodialysis [10], these results cannot be extrapolated to patients with PD because the absorption mechanisms of the peritoneum are different from blood absorption. In addition, peritonitis alters the permeability of the peritoneal membrane, leading to higher systemic absorption of drugs than among PD patients without peritonitis [11]. Blackwell et al. [12] determined serum levels of ampicillin after administering 2 g intraperitoneal ampicillin to six healthy patients undergoing CAPD [12]. On this basis, it would have been expected that the serum levels after application of 2 g ampicillin intraperitoneally in peritonitis would be higher than those determined by Blackwell et al. in healthy patients. However, we detected serum levels of ampicillin comparable to those detected by Blackwell et al. after we administered 4 g intraperitoneal ampicillin.

Therefore, because determining a clear generalized recommendation for the dosage of intraperitoneal ampicillin does not appear to be possible, the dosage for these patients must be determined individually in the context of peritonitis and diuresis. For this purpose, the determination of serum concentrations of ampicillin can be helpful and is recommended.

In this case, adequate serum levels of ampicillin were achieved even when ampicillin was administered intraperitoneally only intermittently for 12 h, and this method of administration also effectively reduced the leukocyte count in the PD effluent. The current ISPD guideline contains no recommendation for intermittent administration of ampicillin, unlike vancomycin or gentamicin; instead, the guideline recommends a maintenance dose administered continuously in all dialysate exchanges [2].

To our knowledge, this is the first report of a case of PD-related peritonitis with *L. monocytogenes* for which serum ampicillin concentrations were monitored for both intravenous and intraperitoneal administration. Even though serum concentrations of ampicillin were clearly sufficient with intravenous administration alone, a permanent reduction of the leukocyte count in the PD effluent was achieved only by combined intravenous and intraperitoneal administration of ampicillin. Adequate serum ampicillin concentrations were also achieved with intraperitoneal application alone. Thus, the ampicillin concentrations reported here provide a good indication of the dosage of intraperitoneal ampicillin to be used to treat PD-related peritonitis. However, pharmacokinetic studies are necessary for a more detailed therapy recommendation.

Peritonitis with *L. monocytogenes* is a serious complication of PD. Recent guidelines provide only an imprecise therapy recommendation regarding the choice of antibiotic, the dosage, and the route of application. This report presents the first case of intraperitoneal treatment of PD-related peritonitis for which serum concentrations of ampicillin were determined.

We found that intraperitoneal administration of ampicillin at the site of infection produced a sufficiently high systemic concentration of the drug, whereas intravenous administration did not adequately treat peritonitis even with very high serum levels of ampicillin. Pharmacokinetic studies are needed for specifying therapy recommendations.

## Data Availability

All relevant data are shown in the case report.

## Abbreviations

BMI: Body mass index
CAPD: Continuous ambulatory peritoneal dialysis
CCPD: Continuous cyclic peritoneal dialysis
CRP: C-reactive protein
EUCAST: European Committee on Antimicrobial Susceptibility Testing
ISPD: International Society for Peritoneal Dialysis
LAA: Left atrial appendage
MIC: Minimal inhibitory concentration
PCT: Procalcitonin
PD: Peritoneal dialysis
